# End-to-end simulation of nanopore sequencing signals with feed-forward transformers

**DOI:** 10.1093/bioinformatics/btae744

**Published:** 2024-12-23

**Authors:** Denis Beslic, Martin Kucklick, Susanne Engelmann, Stephan Fuchs, Bernhard Y Renard, Nils Körber

**Affiliations:** Centre for Artificial Intelligence in Public Health Research, Robert Koch Institute, Berlin 13353, Germany; Institute for Microbiology, Technical University of Braunschweig, Braunschweig 38106, Germany; Microbial Proteomics, Helmholtz Centre for Infection Research (HZI), Braunschweig 38124, Germany; Institute for Microbiology, Technical University of Braunschweig, Braunschweig 38106, Germany; Microbial Proteomics, Helmholtz Centre for Infection Research (HZI), Braunschweig 38124, Germany; Genome Competence Center, Robert Koch Institute, Berlin 13353, Germany; Data Analytics and Computational Statistics, Hasso Plattner Institute, Digital Engineering Faculty, University of Potsdam, Potsdam 14482, Germany; Centre for Artificial Intelligence in Public Health Research, Robert Koch Institute, Berlin 13353, Germany

## Abstract

**Motivation:**

Nanopore sequencing represents a significant advancement in genomics, enabling direct long-read DNA sequencing at the single-molecule level. Accurate simulation of nanopore sequencing signals from nucleotide sequences is crucial for method development and for complementing experimental data. Most existing approaches rely on predefined statistical models, which may not adequately capture the properties of experimental signal data. Furthermore, these simulators were developed for earlier versions of nanopore chemistry, which limits their applicability and adaptability to the latest flow cell data.

**Results:**

To enhance the quality of artificial signals, we introduce *seq2squiggle*, a novel transformer-based, non-autoregressive model designed to generate nanopore sequencing signals from nucleotide sequences. Unlike existing simulators that rely on static k-mer models, our approach learns sequential contextual information from segmented signal data. We benchmark *seq2squiggle* against state-of-the-art simulators on real experimental R9.4.1 and R10.4.1 data, evaluating signal similarity, basecalling accuracy, and variant detection rates. *Seq2squiggle* consistently outperforms existing tools across multiple datasets, demonstrating superior similarity to real data and offering a robust solution for simulating nanopore sequencing signals with the latest flow cell generation.

**Availability and implementation:**

*seq2squiggle* is freely available on GitHub at: github.com/ZKI-PH-ImageAnalysis/seq2squiggle.

## 1 Introduction

Long-read nanopore sequencing has emerged as a transformative technology in the field of genomics, offering rapid and cost-effective DNA sequencing capabilities with applications ranging from fundamental research to clinical diagnostics (Giesselmann 2021). The translocation of analyte molecules through a nanometer-sized pore generates a distinct current signal that represents the physical properties of the molecule inside the pore. These signals are then translated into corresponding DNA sequences (Delahaye and Nicolas 2021). Nanopore sequencing has significantly advanced our ability to detect single nucleotide variations (SNVs) and insertions/deletions (indels), which are crucial for understanding genetic diversity and susceptibility to diseases. SNVs, or single nucleotide polymorphisms, play a pivotal role in genomic diversity and disease susceptibility, while indels involve mutations where nucleotides are either inserted or deleted from the DNA sequence. Accurate detection of these variations is particularly challenging with nanopore sequencing due to potential errors introduced by homopolymer regions, which can lead to false-positive calls (Delahaye and Nicolas 2021, Wang *et al.* 2021). The potential of long-read sequencing has sparked the development of a large variety of methods for basecalling, modification detection, error correction, genome assembly, and detection of structural variations (Amarasinghe *et al.* 2020).

**Figure 1.**
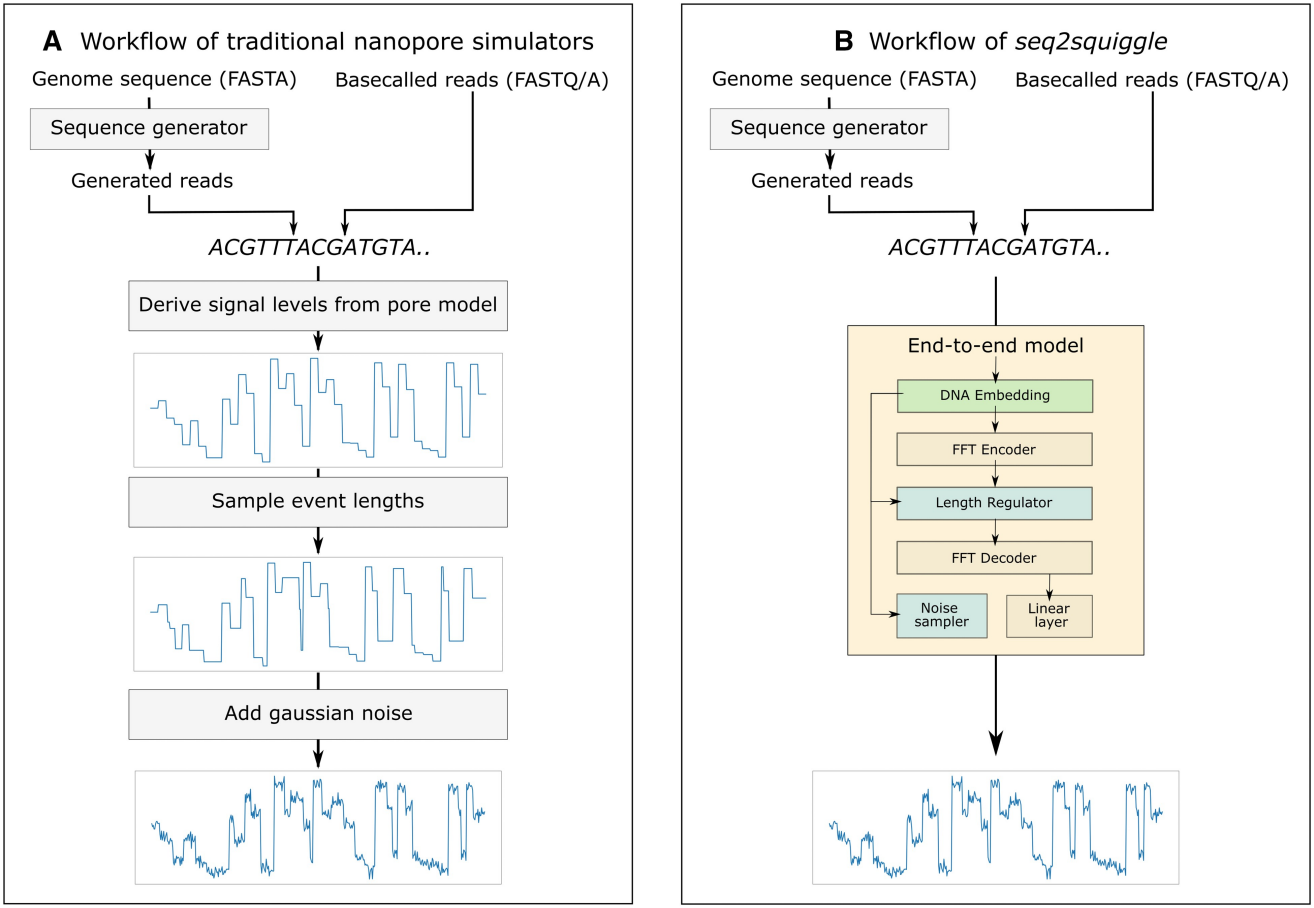
Comparison of nanopore signal simulators with *seq2squiggle*. (A) Traditional nanopore simulators process either read-input (FASTQ/A) or genome-input (FASTA). For genome-input, these simulators first use a sequence generator to produce reads. They then calculate event levels using pre-defined pore models, sample event durations from random distributions, and add Gaussian noise with fixed parameters across all input sequences. While some tools incorporate deep learning for specific sub-modules (e.g. pore models or low-pass filter), these methods are limited to enhancing certain components. (B) In contrast, *seq2squiggle* uses an end-to-end deep learning approach to predict signals directly from nucleotide input, allowing the model to learn signal levels, event durations, and noise distributions from the training data itself.

Simulation of sequencing signals from nucleotide data is crucial to complement experimental nanopore data and to benchmark recently developed methods. It allows researchers to refine experimental protocols, evaluate sequencing performance, and deepen the understanding of the interactions between DNA molecules and nanopores (Li *et al.* 2018). Sequencing simulators such as DeepSimulator (Li *et al.* 2018), NanosigSim (Chen *et al.* 2020), and squigulator (Gamaarachchi *et al.* 2024) generate nanopore sequencing signals using input nucleotide sequences and pre-existing k-mer models. These simulators first calculate the event level of each k-mer based on pore models provided by Oxford Nanopore Technologies (ONT), then sample the duration of each event from a random distribution (e.g. gamma distribution), and finally add Gaussian noise to the signal (Fig. 1A). Although DeepSimulator and NanosigSim incorporate deep learning techniques in their methods, these are limited to specific modules for improvements in pore model accuracy or noise generation. Moreover, these simulators were developed and optimized using the earlier R9.4.1 chemistry and have not been evaluated with the most recent R10.4.1 chemistry, which exhibits a distinct signal profile due to its modified protein pore and two measurement points (Ahsan *et al.* 2024). Of the mentioned tools only squigulator is capable of generating data for the latest pore chemistry.

The reliance on pre-defined pore models and constant statistical assumptions across all sequences poses a risk of inaccuracies in generating in-silico signal data. Teng *et al.* highlighted that the segmentation of raw signals is a critical source of errors in basecalling algorithms (Teng *et al.* 2018), which has led to a paradigm shift in basecalling methodologies toward “end-to-end” deep learning architectures. Applying a similar end-to-end approach to signal simulation offers the potential to enhance the quality of simulated data. These frameworks bypass the segmentation step and directly infer nucleotide sequences from raw signals, improving accuracy, and robustness. However, simulating signals from DNA sequences presents a one-to-many mapping challenge, where each DNA sequence corresponds to a variety of possible target signals of alternative event lengths and noise levels. This inherent complexity introduces unique hurdles in defining appropriate loss functions, designing model architectures capable of capturing intricate relationships between sequences and signals, and identifying suitable evaluation metrics. Overcoming these challenges with deep learning frameworks is crucial for improving the accuracy of artificial nanopore signals and supporting the development and benchmarking of new analytical methods.

In this study, we introduce *seq2squiggle*, an innovative transformer-based simulator designed for nanopore sequencing data. Similar to the trend in basecallers, our objective is to predict raw signals from sequence data without directly relying on pore models. By leveraging feed-forward transformer (FFT) blocks, our model effectively captures broader sequential contexts, enabling the generation of artificial signals that closely resemble experimental observations. Furthermore, *seq2squiggle* includes modules capable of learning event length and amplitude noise distributions from training data (Fig. 1B). Evaluation against multiple experimental R9.4.1. and R10.4.1 datasets reveals that our proposed model outperforms existing tools in generating high-quality simulated reads with higher match rates and Q-scores, while its adjustable noise modules enable approximation of real-world data variability.

## 2 Materials and methods

### 2.1 General workflow overview

In this section, we present a general overview of our proposed simulator *seq2squiggle*, a novel tool designed for simulating nanopore sequencing signals using FFT blocks. Similar to previous simulators, the initial module of our tool is the sequence generator. Given a user-specified reference genome, as well as the desired coverage or number of reads, this module randomly selects starting positions on the genome or contigs to generate sequences that meet the coverage requirements and mimic the length distribution of experimental nanopore reads. *Seq2squiggle* can generate reads from a genome to simulate signals or use experimental reads via the—read-input command. The read length distribution can be influenced by various factors, such as the sample species and experimental setup (Baker *et al.* 2016, Li *et al.* 2018). Given that realistic read-length simulation is complex and not the primary focus of our research, we chose to adopt the read-length distributions previously defined and used in DeepSimulator (Li *et al.* 2018). These include an exponential distribution, a beta distribution, and a mixed gamma distribution, which provide a practical and established framework for our simulator.

To enhance the quality of the simulated signals, *seq2squiggle* avoids the direct use of pore models and instead learns the signal characteristics from segmented signal data. The details of the signal generation using FFTs are described in the next chapter.

The simulated reads are either exported to the community-driven SLOW5 format (Gamaarachchi *et al.* 2022) or the new POD5 format by ONT, enabling seamless integration for basecalling and subsequent analysis. This ensures compatibility with existing nanopore sequencing analysis pipelines and supports comprehensive downstream evaluations.

### 2.2 Model architecture

**Figure 2.**
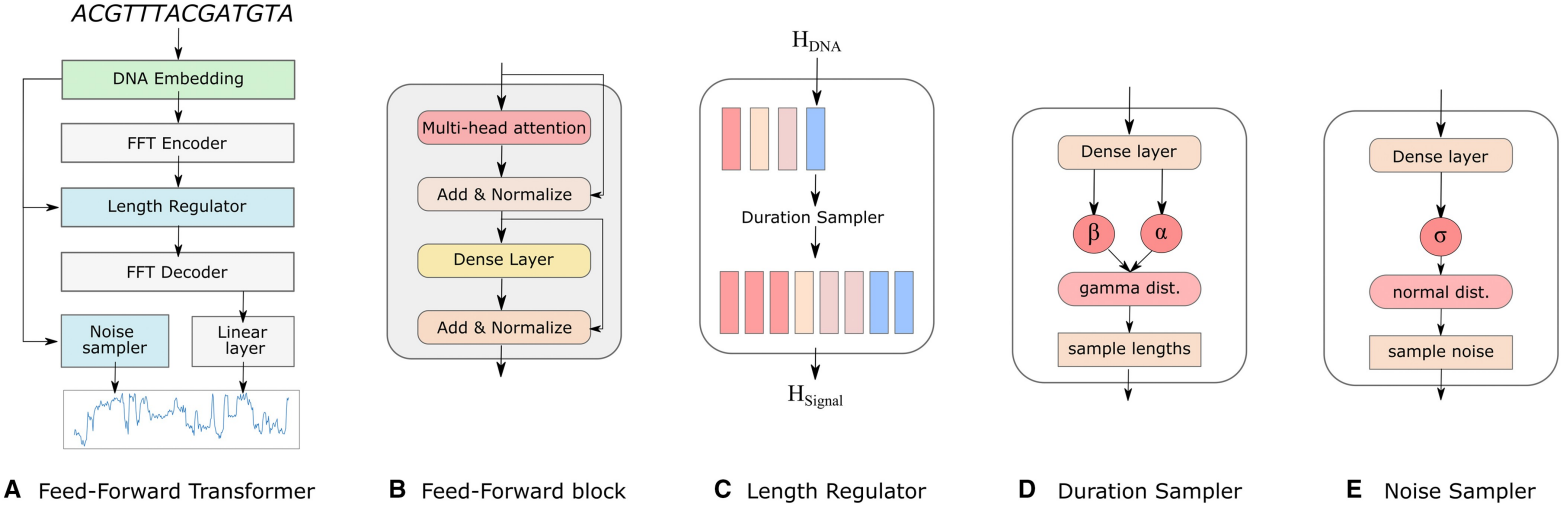
The architecture of *seq2squiggle*. (A) The FFT is the core model architecture, using feed-forward networks to map DNA sequences to nanopore signals. (B) The feed-foward block consists of multi-head attention and dense layers for cross-position information extraction. (C) The length regulator adjusts the hidden states of the DNA sequences to match the predicted event duration of the nanopore signal. (D) The duration sampler uses a gamma distribution to sample the event lengths based on the DNA embeddings. (E) The noise sampler predicts the standard deviation of gaussian noise based on the DNA embeddings and adds it to the signal to create realistic noise patterns.

Our proposed model *seq2squiggle* predicts nanopore sequencing signals using a FFT (Fig. 2A), a deep learning architecture developed and successfully applied for speech synthesis (Ren *et al.* 2019, 2022). Similar to text-to-speech approaches, we map the shorter source sequence (DNA) to the larger target sequence (nanopore signal) for which we used a feed-forward architecture instead of an autoregressive network.

As input for training *seq2squiggle*, we use segmented DNA-to-signal mapping obtained from the *eventalign* output of *uncalled4*, a toolkit for nanopore signal alignment developed by Kovaka *et al.* (2024). The *eventalign* table includes each mapped k-mer sequence, its aligned raw signal data, and its deviation from the ideal pore model value. For each prediction, *seq2squiggle* takes input chunks of 16 overlapping k-mer sequences and maps them to a maximum of 250 signal points. The reference signal is created by concatenating raw signal values from these overlapping k-mers, with shorter signals zero-padded and chunks exceeding 250 signal points filtered out. The model vocabulary includes five symbols: the four DNA bases (“A,” “C,” “G,” “T”) and an empty symbol (“_”).

To generate DNA embeddings, we one-hot encoded each nucleotide within each 16-k-mer chunk, where each nucleotide is represented by a unique binary vector. This one-hot encoding is subsequently flattened and processed through a dense layer that maps the one-hot encoded vector to a higher-dimensional space using a Rectified Linear Unit (ReLU) activation function. Once embedded, the DNA sequences are processed by a FFT encoder, which includes two FFT blocks to capture positional and contextual information across the sequence. Each FFT block consists of a standard multi-head attention mechanism and feed-forward dense layers, allowing the model to capture complex dependencies and relationships within the input sequence (Fig. 2B).

To address the alignment issue between input and target sequences, a length regulator is implemented. This component upscales the hidden states of the DNA sequence according to the event duration of the corresponding nanopore signal (Fig. 2C). Following the length regulator, the upscaled DNA hidden states are processed by an FFT decoder, which consists of two FFT blocks to further align the context with the target nanopore signal.

To infer the duration lengths of each signal, we use a duration sampler consisting of two dense layers with ReLU activation resulting in two scalars α and β (Fig. 2D). These two scalars are used for parameterizing a gamma distribution from which we sample the duration lengths. Our proposed duration sampler takes the sequence embeddings as input for generating event lengths based on a random distribution. The event lengths from the duration sampler are only used in the inference phase since we can use the reference event duration during training.

In addition, we incorporate a noise module consisting of two dense layers with ReLU activation, which predicts the standard deviation *σ* of the signal. Similar to the duration sampler, the prediction is based on the embedded nucleotide sequence, serving as input for the noise module. The predicted standard deviation is then used to sample Gaussian noise distribution, which is subsequently added to the signal after the FFT decoder, contributing to the generation of realistic noise patterns (Fig. 2E).

Hence, *seq2squiggle* incorporates three loss functions, in which *n* denotes the number of samples in a batch and *i* indexes the position within a sample in the *eventalign* table:

1. Signal loss, measured by the mean squared error (MSE), compares the ground truth signal *s*_ref_ with the predicted signal s_pred_:
  $${\text{MSE}}_{\text{signal}}=\frac{1}{n}\sum_{i=1}^{n}{\left(s_{\text{ref},i}-s_{\text{pred},i}\right)}^{2}$$
2. Duration loss utilizes the negative log-likelihood (NLL) to observe the reference event lengths l_data_ given the parameterized gamma distribution with predicted shape *α* and rate *β*. The derived parameters *α* and *β* are predicted from the DNA embeddings using a dense layer. To ensure that the scale of this loss is comparable to the other loss functions, it is adjusted by the factor τ = 0.0005.
  $${\text{NLL}}_{\text{duration}}=\tau \times \frac{1}{n}\sum_{i=1}^{n}\left[-\log P\left(l_{\text{data},i}|\alpha ,\beta \right)\right]$$
3. Noise loss, also measured by the MSE, compares the standard deviation of the real data *σ*_ref_, obtained from the *eventalign* table, with the predicted standard deviation *σ*_pred_ generated by the noise sampler, thereby capturing the variability in the real data.
  $${\text{MSE}}_{\text{noise}}=\frac{1}{n}\sum_{i=1}^{n}{\left(\sigma_{\text{ref},i}-\sigma_{\text{pred},i}\right)}^{2}$$

To optimize the model, the three loss functions are combined into a total loss via weighted summation. Summing multiple loss functions, a common approach in multi-task learning (Liang and Zhang 2021), ensures a balanced optimization process by integrating different aspects of model performance, thereby allowing all objectives to be addressed simultaneously during training:

$$\begin{array}{c} {\text{Loss}}_{\text{total}}={\text{MSE}}_{\text{signal}}\left(s_{\text{ref}},s_{\text{pred}}\right)\\ +{\text{NLL}}_{\text{duration}}\left(l_{\text{data}},\alpha ,\beta \right)+{\text{MSE}}_{\text{noise}}\left(\sigma_{\text{ref}},\sigma_{\text{pred}}\right) \end{array}$$

### 2.3 Experimental setup

#### 2.3.1 Datasets for training and evaluation

For our experiments, we used five publicly available DNA datasets sequenced with different sequencing kits, flow cell chemistries and sampling rates. These included the R10.4.1 Human HCT116 and *Drosophila melanogaster* datasets, both sequenced with Kit14 chemistry at a 4 kHz sampling rate, an *Escherichia coli* dataset sequenced with R10.4.1 Kit14 chemistry at a 5 kHz sampling rate, and the R9.4.1 NA12878 Human and SARS-CoV-2 SP1 datasets, both sequenced with Kit10 chemistry at a 4 kHz sampling rate (Supplementary Table S1).

All datasets were converted to the POD5 format when necessary using blue-crab (Samarakoon *et al.* 2023) or the POD5 library. Basecalling was performed with dorado (v0.8.0) using the models specified in Supplementary Table S1, and the reads were then aligned with minimap2 (v2.26) to reference sequences indicated therein. To segment the reads and establish a mapping between the DNA sequence and the nanopore signal, we used the *eventalign* option from uncalled4 (v4.1).

For training the R10.4.1 model of *seq2squiggle*, we used 198 893 973 chunks derived from 1 424 222 reads of chromosome 2, 3, and 4 (chr2-4) of the Human HCT116 dataset. For validation, we used 100 000 chunks from 56 reads of chromosome 22 (chr22) from the same dataset. For evaluation, we used 100 000 reads from chromosome 1 (chr1) of the Human HCT116 dataset, 100 000 reads from the *D.melanogaster* dataset, and 100 000 reads from the *E.coli* dataset. Similarly, the R9.4.1 model of *seq2squiggle* was trained using 62 486 122 chunks from 108 649 reads of chromosomes 2, 3, and 4 (chr2-4) from the Human NA13878 dataset, validated on 100 000 chunks from chromosome 22 (chr22). For evaluation, we used 50 000 reads from chromosome 1 (chr1) of the Human NA12878 dataset, and 50 000 reads from the SARS-CoV-2 SP1 dataset. This approach allowed us to comprehensively assess the performance of *seq2squiggle* across different genomic contexts. Details on the parameters used during training are described in Supplementary Data.

#### 2.3.2 Evaluation methods

We evaluated the performance of *seq2squiggle* and squigulator through two modes: Read-mode and Genome-mode.

**Read-mode**: Here, signals were simulated based on experimentally basecalled reads (FASTQ/A data). This approach enabled a direct comparison between simulators and the experimental data using the same set of reads, focusing on signal similarity and basecalling metrics. We used dynamic time warping (DTW) to assess signal similarity because it effectively handles variations in signal length and temporal alignment, which are common in nanopore sequencing data. We used mrmsdtw (Pratzlich *et al.* 2016), a memory-efficient implementation of DTW provided by the linmdtw package (Tralie and Dempsey 2020). Mrmsdtw offers reduced memory usage compared to the traditional DTW implementation, making it suitable for handling large-scale nanopore signal comparisons. We normalized the mrmsdtw values to account for read length differences and limited our analysis to 2000 reads to manage computational demands.

**Genome-mode**: In this mode, simulators generated reads from the same input genome (FASTA data). Thereafter, signals are simulated based on these generated reads. This allowed us to evaluate the simulators' ability to replicate the quality of real nanopore sequencing reads. Key metrics included successful alignment rate, alignment ratio, match rate, mismatch rate, insertion rate, deletion rate, and the AUC of the average match rate, as described previously (Pagès-Gallego and De Ridder 2023).

In addition, we examined the variant detection rate by integrating high-confidence NA12878 variants from Genome in a Bottle (v3.3.2) into chromosome 22 of the human reference genome, similar to previous work by Gamaarachchi *et al.* (Gamaarachchi *et al.* 2024). These modified sequences were simulated using two tools, *seq2squiggle* and squigulator, on R10.4.1 chemistry, generating 250 000 reads with each simulator. In addition, we generated 50 000 reads on R9.4.1 chemistry using *seq2squiggle*, squigulator, and two configurations of DeepSimulator (context-dependent and context-independent). All simulated reads were subsequently basecalled and aligned to the hg38 reference genome, following a similar procedure performed by Gamaarachchi *et al.* (2024). Variant calling was performed using Clair3 (Zheng *et al.* 2022) and the results were evaluated using RTGtools (Cleary *et al.* 2014) against the integrated high-confidence variants. Detailed commands and additional information are provided in the Supplementary Data.

## 3 Results

### 3.1 Signal similarity and basecalling accuracy

To evaluate the performance of *seq2squiggle*, we conducted a series of experiments on both R9.4.1 and R10.4.1 datasets, comparing our tool against other simulators and experimental data. Squigulator was initially chosen as a baseline due to its superior performance on R9.4.1 data over existing simulators, such as DeepSimulator, as reported by Gamaarachchi *et al.* (2024), and because it is currently the only simulator supporting R10.4.1 chemistry. For our extended analysis on R9.4.1 chemistry, we also compared *seq2squiggle* with DeepSimulator in both context-dependent and context-independent modes.

Initially, on R10.4.1 data, we compared *seq2squiggle* and squigulator both simulators in Read-mode, where they simulated the same set of experimental input reads. For this comparison, we evaluated both tools in their default mode, which added noise to both the event-length and signal-amplitude domains. In Read-mode, we compared the DTW distance of each simulated read to the corresponding real experimental read (Supplementary Fig. S1). The results indicated that reads generated by squigulator exhibited a slightly larger DTW deviation (median 10.01) from the real experimental reads compared to those generated by *seq2squiggle* (median 9.78). This represents a small but notable increase in DTW deviation for squigulator. However, while DTW distance provides an initial measure of signal similarity, it does not differentiate between DNA bases and thus does not fully capture the impact on downstream analysis. DTW measures overall signal similarity but cannot account for how deviations in the signal might affect the accuracy of basecalling. For certain DNA bases, these deviations might be tolerable and still result in correct basecalls, whereas for others, they could significantly alter the basecalling outcome. Such nuances are not reflected by DTW alone and can only be understood through downstream analysis.

**Figure 3.**
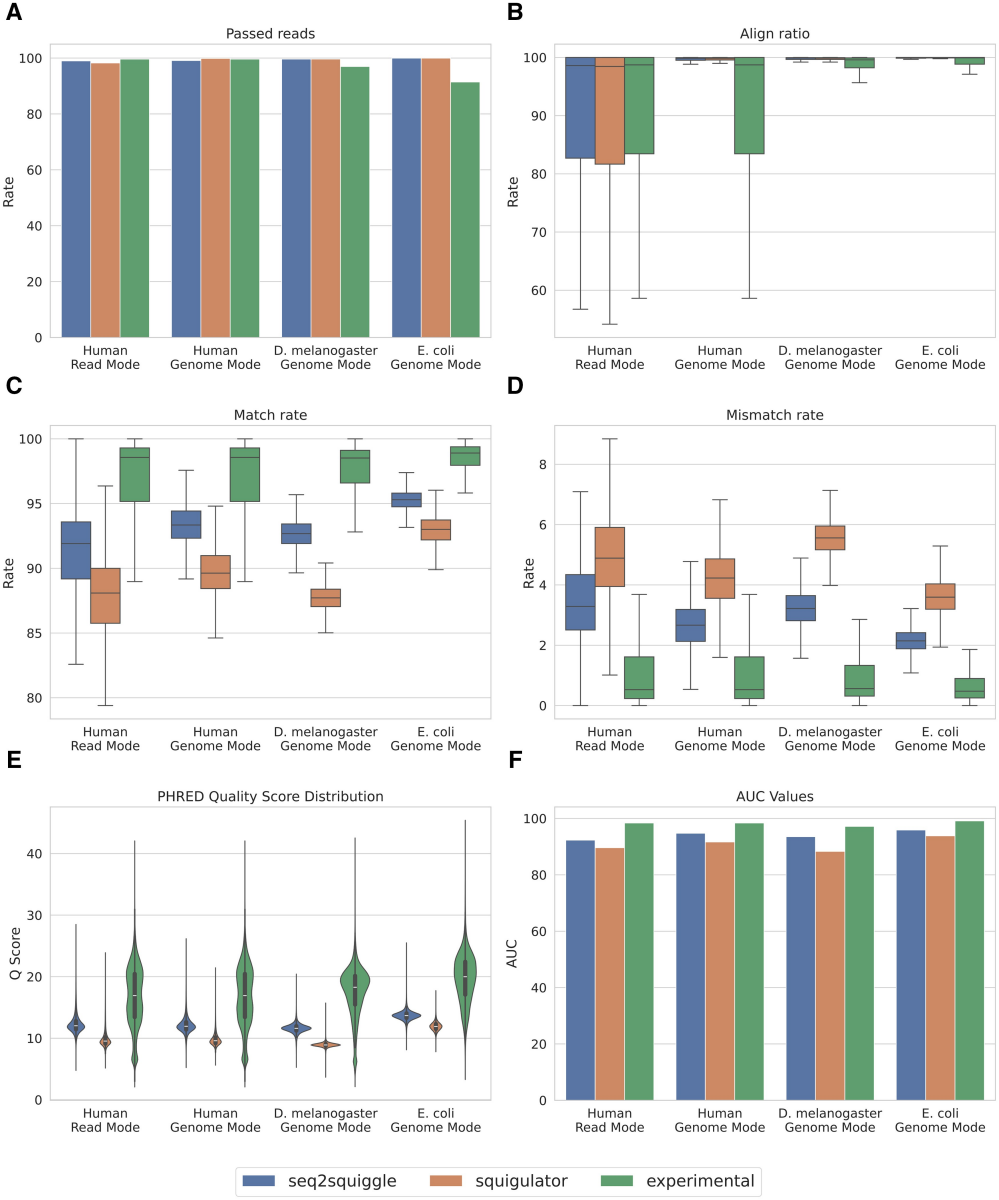
Performance comparison of *seq2squiggle*, squigulator, and experimental data across multiple R.10.4.1 datasets and several performance metrics. (A) Proportion of successfully aligned reads to the reference genome. (B) Distribution of aligned bases to the reference genome. (C) Distribution of match rates. (D) Distribution of mismatch rates. (E) Distribution of PHRED quality scores (F) Area under the curve (AUC) values for match rates sorted by average PHRED scores.

To evaluate the practical significance of these deviations on R10.4.1 data, we extended our comparison to basecalling accuracy and alignment metrics in both Read-mode and Genome-mode using three publicly available datasets. First, we compared the rate of successfully aligned reads and the alignment ratio (Fig. 3A and B). Here, *seq2squiggle’s* reads showed a higher alignment ratio and successful alignments in both Read-mode and Genome-mode. As expected, the alignment ratio in Genome-mode was higher for both simulators since reads were generated directly from the target genome. In Read-mode, however, the signals were generated from basecalled experimental reads, which may contain basecalling errors that impact the alignment accuracy. Our proposed model generated reads with an overall higher median match rate of 91.91 (human Read-mode) to 95.29 (*E.coli* Genome-mode) across all datasets (Fig. 3C). In contrast, squigulator showed a lower match rate of 87.71–93.00. Further, *seq2squiggle* showed a lower mismatch rate (Fig. 3D), a lower deletion rate, and a lower insertion rate (Supplementary Fig. S2) compared to squigulator. Notably, the differences in insertion rates were smaller compared to the differences in mismatch and deletion rates. Reads generated by *seq2squiggle* exhibited an overall higher median PHRED score of 11.61–13.67 while squigulator generated reads with a median Q-score of 8.91–11.91 (Fig. 3E). Finally, we plotted the AUC of the average match rate of the reads sorted by the average PHRED score (Supplementary Fig. S3). Again, *seq2squiggle* showed higher AUC values across all datasets compared to squigulator (Fig. 3F). We also compared the read length distribution of both tools with the experimental data (Supplementary Fig. S2D). Here, both simulators exhibited realistic read length distributions, although variations were observed due to factors such as the species being sequenced, the type of sequencing device used, and other experimental parameters.

For the R9.4.1 data, we compared *seq2squiggle*, squigulator, and DeepSimulator using the same basecalling performance metrics on two publicly available datasets. *Seq2squiggle* consistently demonstrated superior performance, achieving the highest median match rate and the lowest mismatch and deletion rates across all simulators, reflecting an overall higher simulated read quality (Supplementary Fig. S10). Its reads also showed higher median PHRED scores, indicating better overall read quality. Notably, both *seq2squiggle* and squigulator, in their default configurations, generated reads with slightly higher match rates and quality scores than those observed in experimental data. However, both tools offer adjustable noise parameters, enabling users to fine-tune simulated reads to more closely reflect real-world error profiles.

### 3.2 Detection of SNVs and indels

In this section, we present the results of our analysis of SNVs and insertions/deletions (indels) using simulated data. We simulated 250 000 reads from chromosome 22 of the human reference genome for R10.4.1 data, and 50 000 reads for R9.4.1 data, using *seq2squiggle*, squigulator, and DeepSimulator in both context-dependent and context-independent modes. The larger dataset for R10.4.1 was generated to account for its lower average match rate of simulated reads compared to R9.4.1, ensuring sufficient data for a comprehensive evaluation.

**Figure 4.**
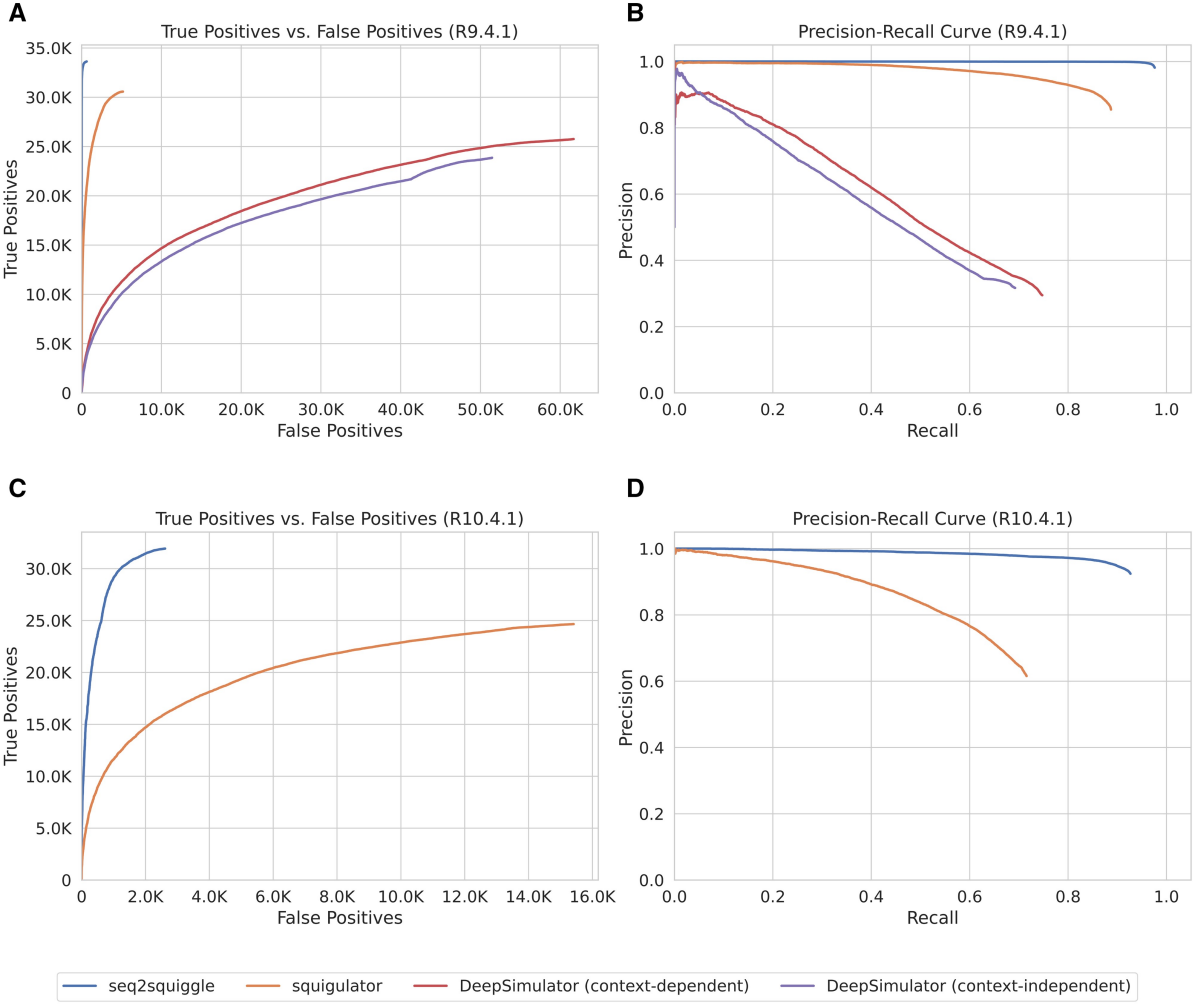
Evaluation of SNP detection accuracy by Clair3 comparing *seq2squiggle*, squigulator, DeepSimulator in context-dependent mode and DeepSimulator in context-independent mode. (A) ROC curve depicting the trade-off between false positives and true positives on R9.4.1 data. (B) Precision-Recall curve showing precision and recall performance on R9.4.1 data. (C) ROC curve for SNP detection on R10.4.1 data. (D) Precision-Recall curve for SNP detection on R10.4.1 data.

Reads generated with *seq2squiggle* showed a higher number of true-positive SNVs, along with an overall higher recall and precision compared to squigulator and DeepSimulator (Fig. 4). However, upon examining the performance of Indel variants on R10.4.1 data (Supplementary Figs S6 and S7), *seq2squiggle* and squigulator both displayed relatively high false-positive rates. The challenges in detecting small Indels with nanopore sequencing, attributed to homopolymer-induced errors, were further exacerbated by the insertion and deletion rates observed in both *seq2squiggle* and squigulator (Supplementary Fig. S2). Hence, the average match rate in homopolymer regions for *seq2squiggle* and squigulator was generally lower compared to experimental data (Supplementary Fig. S8).

Furthermore, we inspected the number of false-positive SNV and Indel calls on R10.4.1 data across specific genomic regions, specifically homopolymer regions (defined as sequences with at least five consecutive identical bases), tandem repeat regions [defined using Tandem Repeat Finder (Benson 1999)], and regions without homopolymers or tandem repeat elements (Supplementary Fig. S9). Our analysis revealed that homopolymer regions yielded a lower percentage of true positives for squigulator and *seq2squiggle*. However, *seq2squiggle* demonstrated a higher number of true-positive variant calls across all three regions.

### 3.3 Influence of noise on performance

We examined the impact of noise parameters and modules on the basecalling performance, a topic previously noted by the authors of squigulator who observed differing effects of noise in the time and amplitude domains on basecalling performance. It was found that higher noise levels in event lengths reduced the accuracy of basecalling. However, intriguingly, optimal basecalling performance was not achieved when no additional amplitude noise was introduced. This observation can be attributed to the basecaller's training on experimental data, which inherently contains noise in the amplitude domain (Gamaarachchi *et al.* 2024).

Here, we conducted similar experiments, where we executed *seq2squiggle* and squigulator across four noise modes on R10.4.1 data: (i) no noise in the amplitude or event-length domain, (ii) noise in the amplitude domain only, (iii) noise in the event-length domain only, and (iv) noise in both the amplitude and event-length domains. These experiments were performed using a subset of 50 000 reads from the *D.melanogaster* dataset. Consistent with prior research, we observed that the incorporation of noise in the amplitude domain enhanced the average match rate compared to noise-free signal simulation in both *seq2squiggle* and squigulator (Supplementary Figs S4 and S5). For *seq2squiggle*, the AUC value increased from 85.41 to 93.93, while for squigulator, it increased from 76.26 to 90.70. Introducing additional noise in the event-length domain resulted in a reduced average match rate, indicating that varying event durations did not significantly aid in distinguishing different DNA bases during basecalling. Introducing duration-noise involved balancing the generation of realistic signals with a slight compromise in performance. Comparing the AUC value of *seq2squiggle* and squigulator, we observed that *seq2squiggle* outperformed squigulator across all four noise modes based on the AUC value (Table 1).

**Table 1.** Benchmark Area under the curve (AUC) values of match rates sorted by PHRED scores for *seq2squiggle* and squigulator in four different noise modes on R10.4.1 data: (1) no noise in amplitude or event-length domain, (2) noise in amplitude domain only, (3) noise in event-length domain only, and (4) noise in both amplitude and event-length domains.

Noise in amplitude domain	Noise in event-length domain	AUC values of *seq2squiggle*	AUC values of squigulator
		85.41	76.26
**✓**		93.93	90.70
	**✓**	83.71	73.15
**✓**	**✓**	93.24	88.14

Furthermore, we modified *seq2squiggle* by replacing both the duration sampler and the noise sampler with static normal distributions (mean = 9.0, SD = 4.0 for length, and mean = 0.0, SD = 1.0 for noise), in accordance to the noise sampling method used in squigulator. This adjustment allowed us to compare the benefits of using duration and noise modules that learned distributions based on DNA sequences, rather than relying on predefined statistical models. Our findings indicated that the noise module in *seq2squiggle* generated signals with a slightly higher match rate (Supplementary Table S4), suggesting that our approach better captured noise patterns that resembled real experimental data. However, the learned duration module resulted in a slightly lower match rate, highlighting that learned variations in event lengths might not enhance performance as effectively as amplitude noise.

In addition, we also compared the median match rate of *seq2squiggle* and squigulator for varying noise degrees in the amplitude domain and event-length domain (Supplementary Figs S12 and S13). For a fair comparison, we again replaced the duration sampler with static noise distributions. As anticipated, higher event-length noise slightly reduced performance for both simulators. Interestingly, our results revealed that for squigulator, the optimal amplitude standard deviation was around 1.0, while for *seq2squiggle*, the optimal value was closer to 2.0. Furthermore, *seq2squiggle* consistently demonstrated a higher median match rate under the same noise conditions, highlighting the advantages of its FFT-based signal generation approach.

## 4 Discussion

In this study, we introduced *seq2squiggle*, an innovative simulator designed for nanopore sequencing data. Unlike existing simulators, our model leverages a FFT architecture to learn the intricate relationship between the input DNA sequence and the corresponding nanopore signal sequence directly from the training data. This approach avoids the direct need for pre-defined statistical models and pore models, which are commonly used in other simulators. By avoiding these models, *seq2squiggle* mitigates potential biases and inaccuracies that can arise from relying on static statistical assumptions.

Our experiments have demonstrated that *seq2squiggle* surpassed existing simulators in generating signals that closely resemble real nanopore sequencing data, achieving higher match rates, PHRED scores, SNP detection rates, and other critical performance metrics on R9.4.1 and R10.4.1 datasets of various species. Despite these advancements, discrepancies between simulated and experimental R10.4.1 data still persist, possibly due to sub-optimal segmentation and the higher complexity of R10.4.1 data compared to R9.4.1 data.

In future work, we aim to improve the architecture of *seq2squiggle* to further increase the basecalling accuracy of simulated reads, aiming for closer alignment with real-world data. Although we currently use modules in the amplitude and event-length domains to sample noise, we make certain assumptions about the noise distributions, such as normal and gamma distributions. Recent innovations in speech synthesis (Huang *et al.* 2022, Zhang *et al.* 2023), particularly the integration of diffusion modules, show promise for advancing our model. By incorporating more sophisticated noise modeling techniques and leveraging diffusion-based approaches, we anticipate further improvements in the quality of our simulated nanopore sequencing signals. In addition, we intend to explore the use of *seq2squiggle* for detecting DNA methylations, expanding its utility in epigenetic studies. Another promising avenue is simulating ONT direct-RNA sequencing (DRS) data, which has become increasingly important in recent years. Although *seq2squiggle* is currently optimized for DNA sequencing, its architecture can be adapted to generate realistic RNA signals by retraining the model on DRS datasets. In future work, we aim to focus on this aspect, allowing *seq2squiggle* to support RNA sequencing applications, which would provide valuable benchmarks and insights for DRS-based research.

It is important to note that *seq2squiggle* has higher runtime and memory consumption compared to squigulator. This is due to its deep learning-based approach, which, while resource-intensive, results in significantly higher quality of the generated signals. The trade-off between computational resources and accuracy is a common challenge in deep learning applications, but the improved performance of *seq2squiggle* justifies its use for applications requiring realistic nanopore signals. Further, its non-autoregressive architecture keeps the runtime in a manageable timeframe to conduct large-scale simulations. Moreover, *seq2squiggle* supports retraining on new datasets or pore chemistries, providing researchers with the flexibility to adapt the model to evolving sequencing platforms. While this capability requires additional computational resources, as detailed in the Supplementary Data, it ensures that *seq2squiggle* remains applicable for future sequencing advancements.

In summary, *seq2squiggle* represents a significant advancement in the simulation of nanopore sequencing data and provides a powerful tool for the nanopore community. Its ability to generate realistic nanopore signals for the latest flow cell generation has great potential for a variety of applications, including method development, benchmarking, and comprehensive genomic analysis.

## Supplementary Material

btae744_Supplementary_Data

## Data Availability

The benchmarking and results can be accessed via Figshare (doi.org/10.6084/m9.figshare.26539696). The benchmarking workflow is available at GitHub (https://github.com/ZKI-PH-ImageAnalysis/seq2squiggle-benchmark).
