# Supplementary material for: End-to-end simulation of nanopore sequencing signals with feed-forward transformers
Source: Bioinformatics. 2024 Dec 23;41(1):btae744. doi: 10.1093/bioinformatics/btae744 (PMC11729726; doi:10.1093/bioinformatics/btae744)
Supplement: btae744_Supplementary_Data [file btae744_supplementary_data.docx]

## Supplementary Data

## S1: Training procedure and required resources

Our model architecture features two FFT blocks on both the DNA-encoder and signal-decoder sides, with hyperparameters set as follows: DNA embeddings, self-attention hidden size, and other hidden dimensions all set to 64; feed-forward projection size set to 256; and 8 attention heads (Supplementary Table 2). The model was trained on 8 NVIDIA A100 GPUs with a batch size of 512 DNA-signal chunks. The training process took approximately 54 hours, requiring a maximum of 50GB of memory and utilizing 128 compute nodes. We employed the Adam optimizer with parameters β1 = 0.9, β2 = 0.98, and ε = 10e−8. A dropout rate of 0.1 and a gradient clipping-by-norm of 1.0 were applied. The learning rate schedule included a linear warm-up (1% ratio) followed by cosine decay, with a maximum learning rate of 0.0005. The model was trained for 20 epochs.

Full details, including used basecalling models, processing parameters, reference sequences, and exact commands, are available in the benchmarking workflow configuration file (<https://github.com/ZKI-PH-ImageAnalysis/seq2squiggle-benchmark>).

## S2: Considerations for re-training the model

The simulation capabilities of *seq2squiggle* are influenced by the training data, particularly factors such as number of modified sites and noise level, which are intrinsic to the specific characteristics of the sequencing platform and the sample conditions. Given these dependencies, users may wonder if they should retrain the model using their own datasets when generating data.

While the variability in training data can affect outcomes, the preprocessing and segmentation performed with *uncalled4* or other segmentation tools can mitigate these effects. Our results have demonstrated the model's robustness in simulating reads across various species, indicating that for most typical applications, the existing model provides a reliable approximation without necessitating retraining.

However, for specialized applications—such as those focusing on species-specific features or methylation research—users may find that retraining the model could be advantageous. It is essential to note that this process demands additional computational resources and time for both preprocessing and training. Users should also consider that reducing the training data size and the number of model parameters can significantly accelerate the training process, as can leveraging a larger number of GPUs.

##


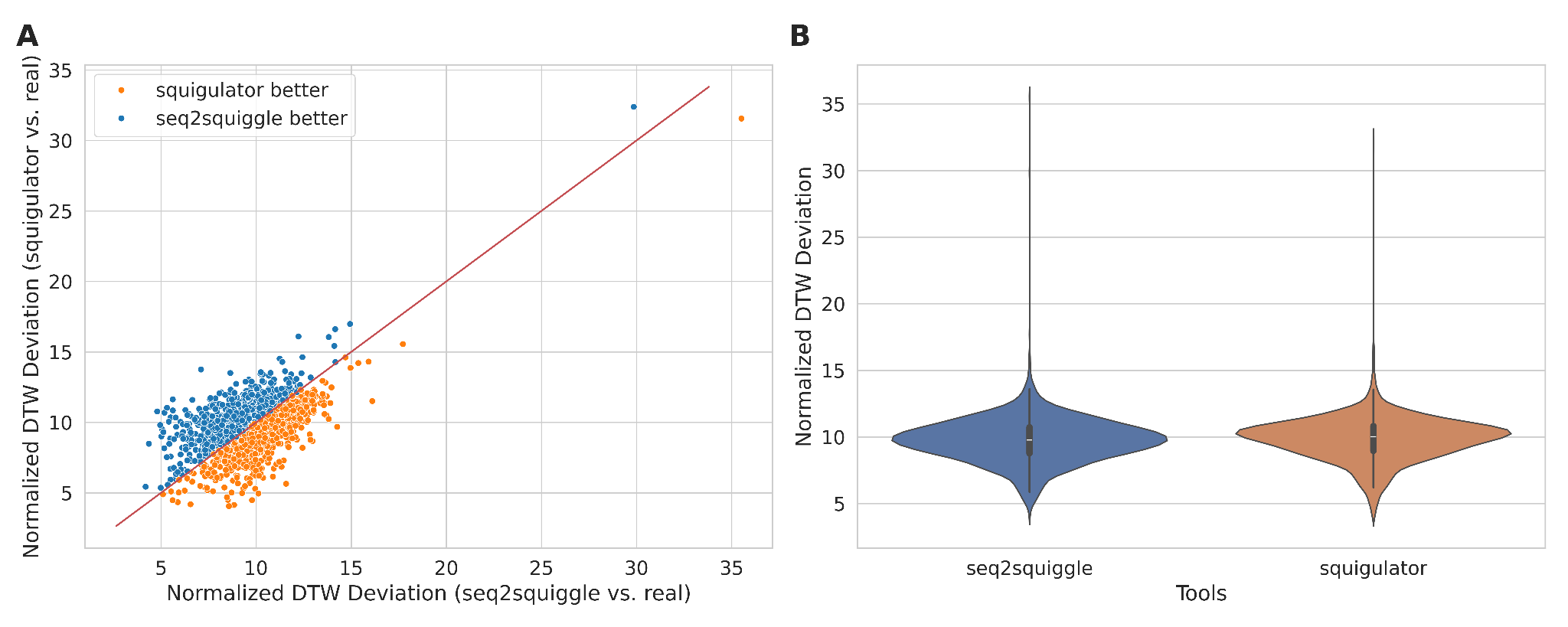


Supplementary Figure 1. Comparison of DTW deviations between the simulators and the experimental data in human read mode of R10.4.1 data. (A) Scatterplot illustrating the DTW deviation between simulated and real signals. Each point represents a read, displaying the DTW deviation of seq2squiggle (X-axis) and squigulator (Y-axis) relative to the real data. Points above the red diagonal line indicate *seq2squiggle* exhibits a smaller DTW deviation compared to squigulator, and vice versa. (B) Violin plot depicting the distribution of DTW deviations between both simulators and real data.


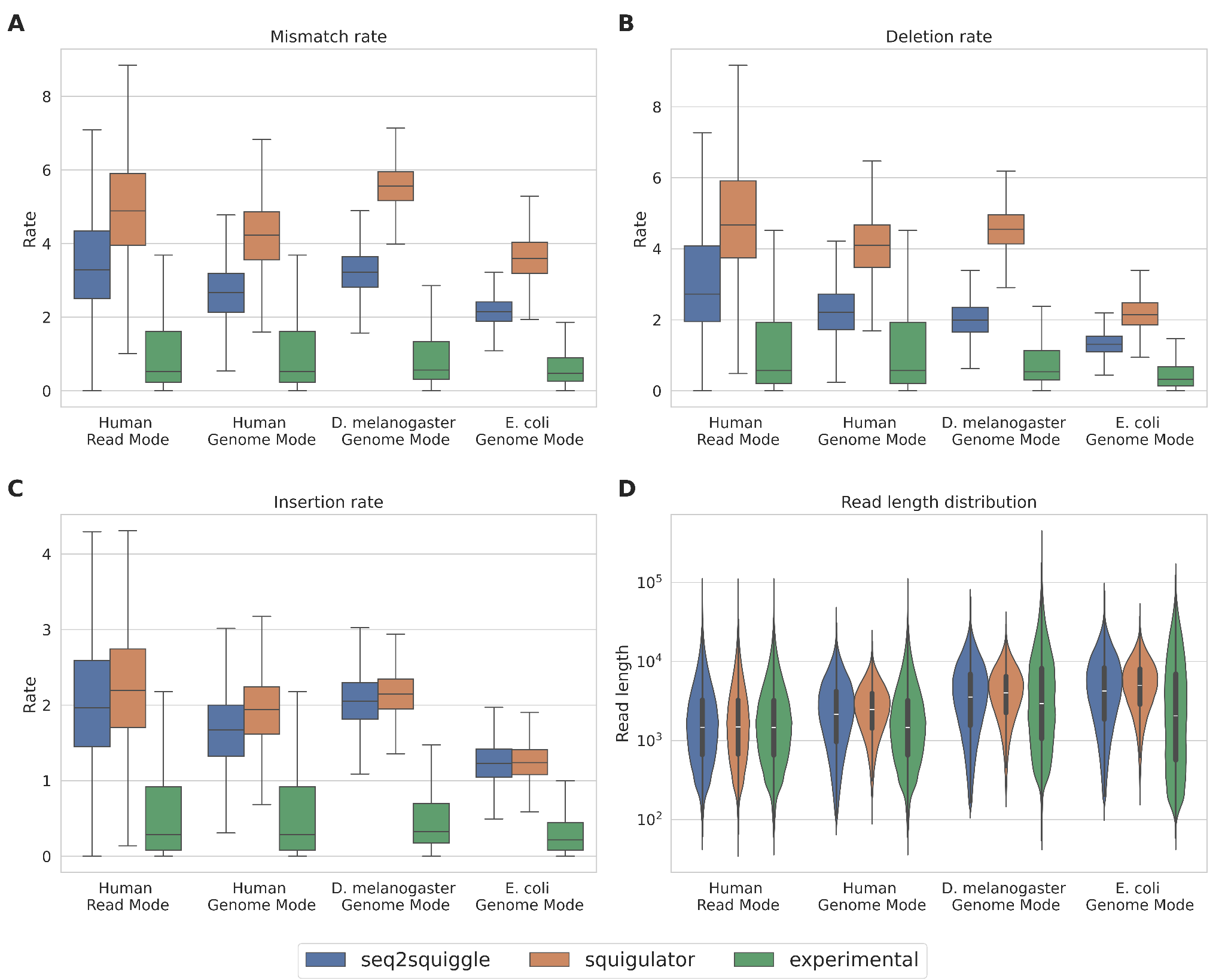


Supplementary Figure 2. Performance comparison of seq2squiggle (blue), squigulator (orange), and experimental data (green) across multiple R10.4.1. datasets and several performance metrics. (A) Distribution of mismatch rates. (B) Distribution of deletion rates. (C) Distribution of insertion rates. (D) Distribution of read lengths.


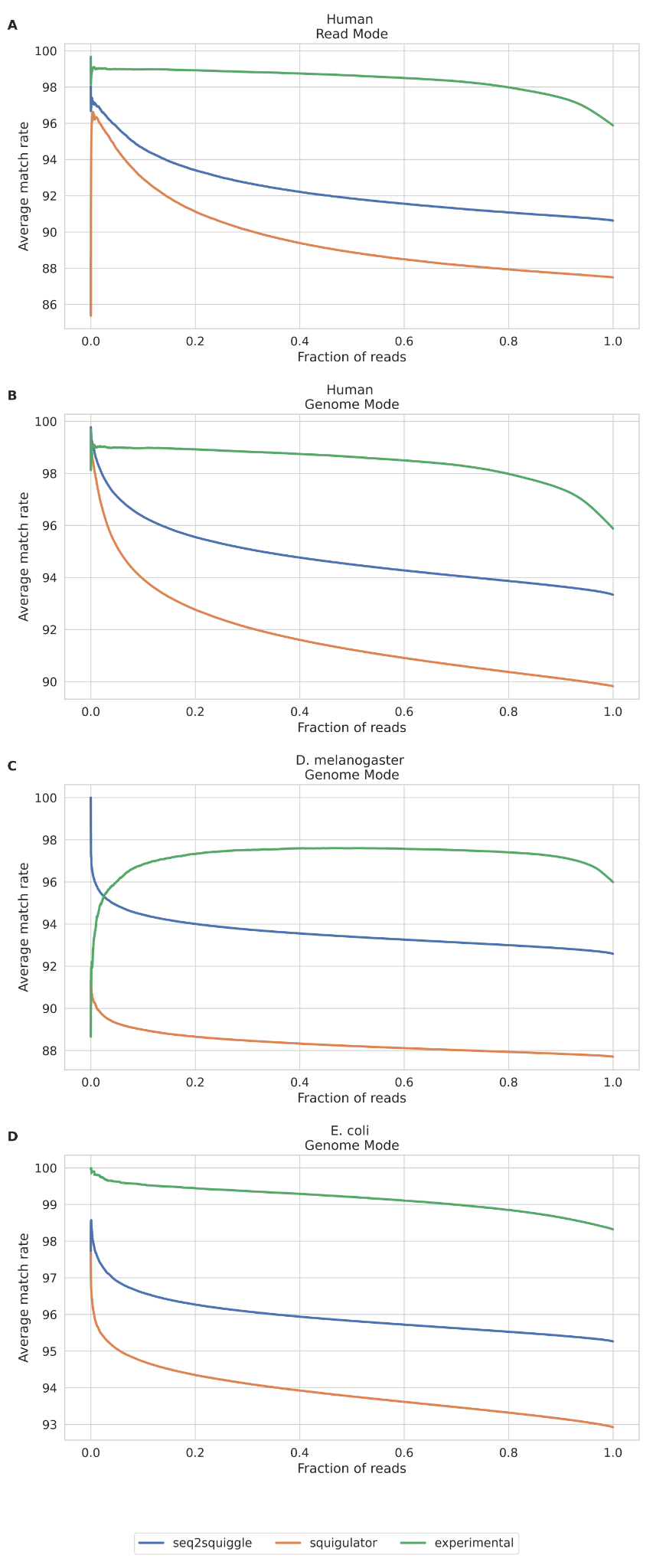


Supplementary Figure 3. AUC of match rate sorted by average PHRED score for seq2squiggle (blue), squigulator (orange), and experimental data (green) across multiple R10.4.1 datasets.


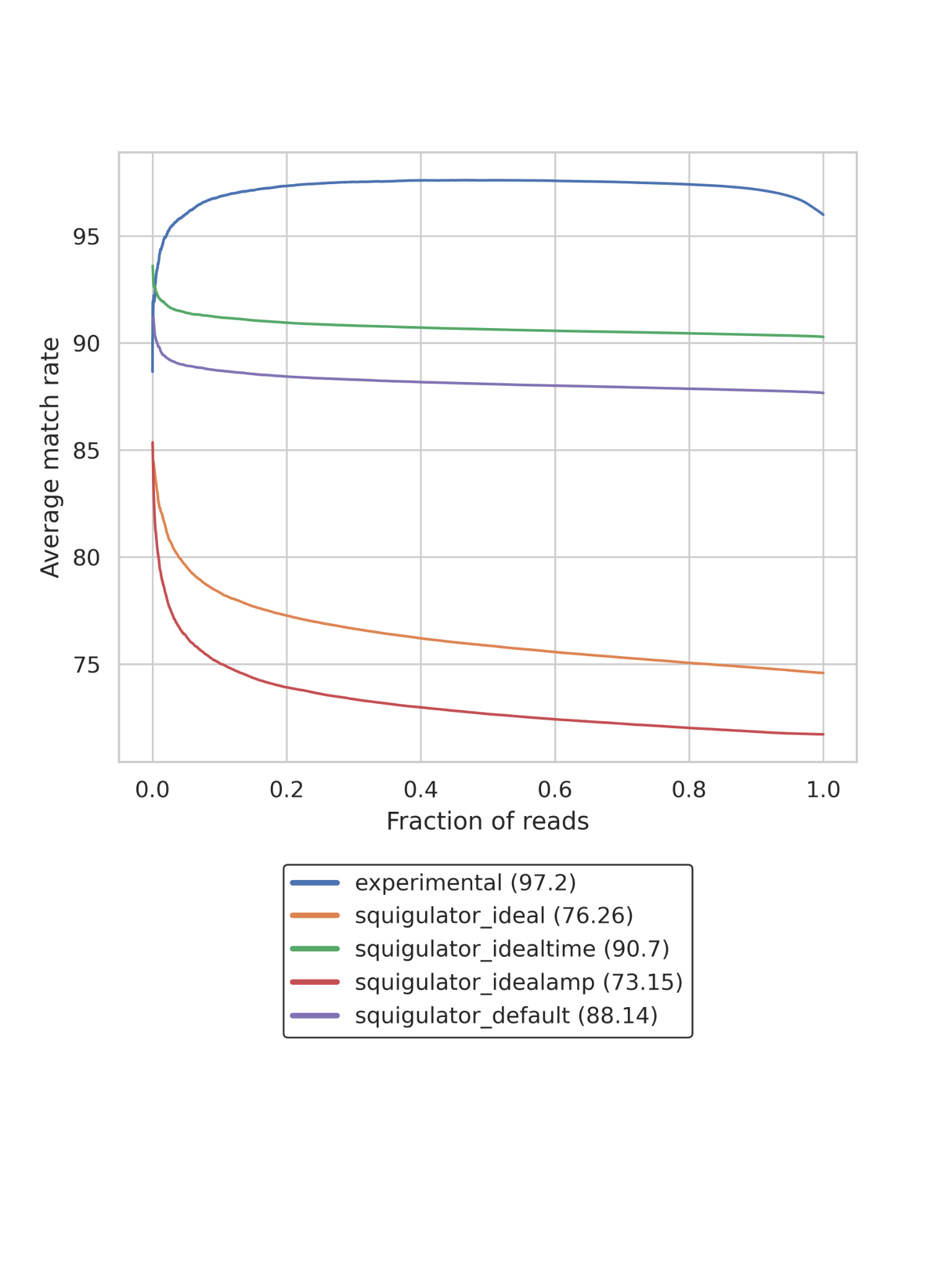


Supplementary Figure 4. AUC of match rate sorted by average PHRED score using different noise modes for squigulator on the R10.4.1 D.melanogaster dataset.


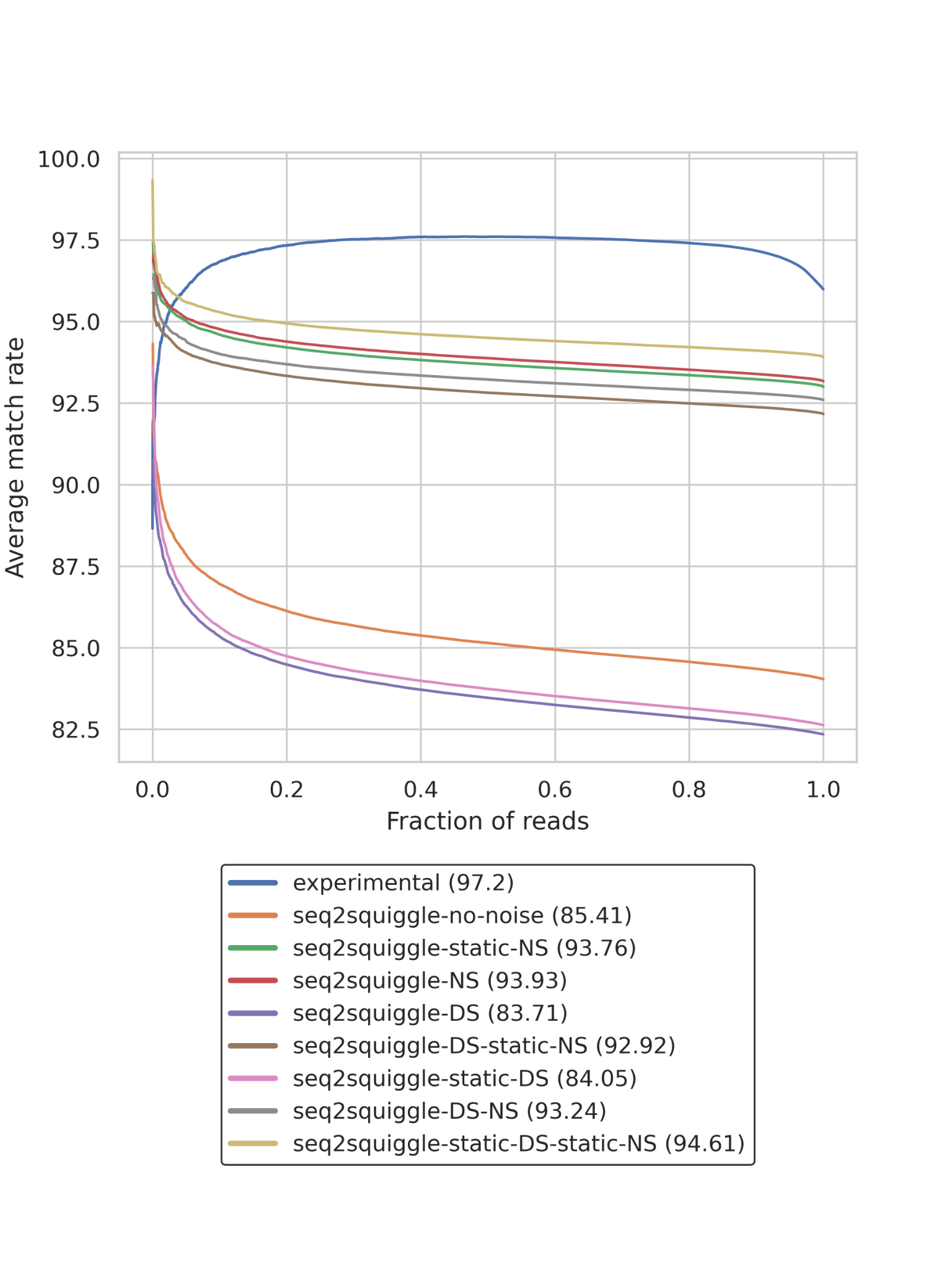


Supplementary Figure 5. AUC of match rate sorted by average PHRED score using various noise modes for *seq2squiggle* on the R10.4.1 D.melanogaster dataset. The noise modes include manual Noise Sampling with static distribution (static NS), Noise Sampling with Noise Sampler (NS), manual duration sampling with static distribution (static DS) and duration sampling with Duration Sampler (DS).


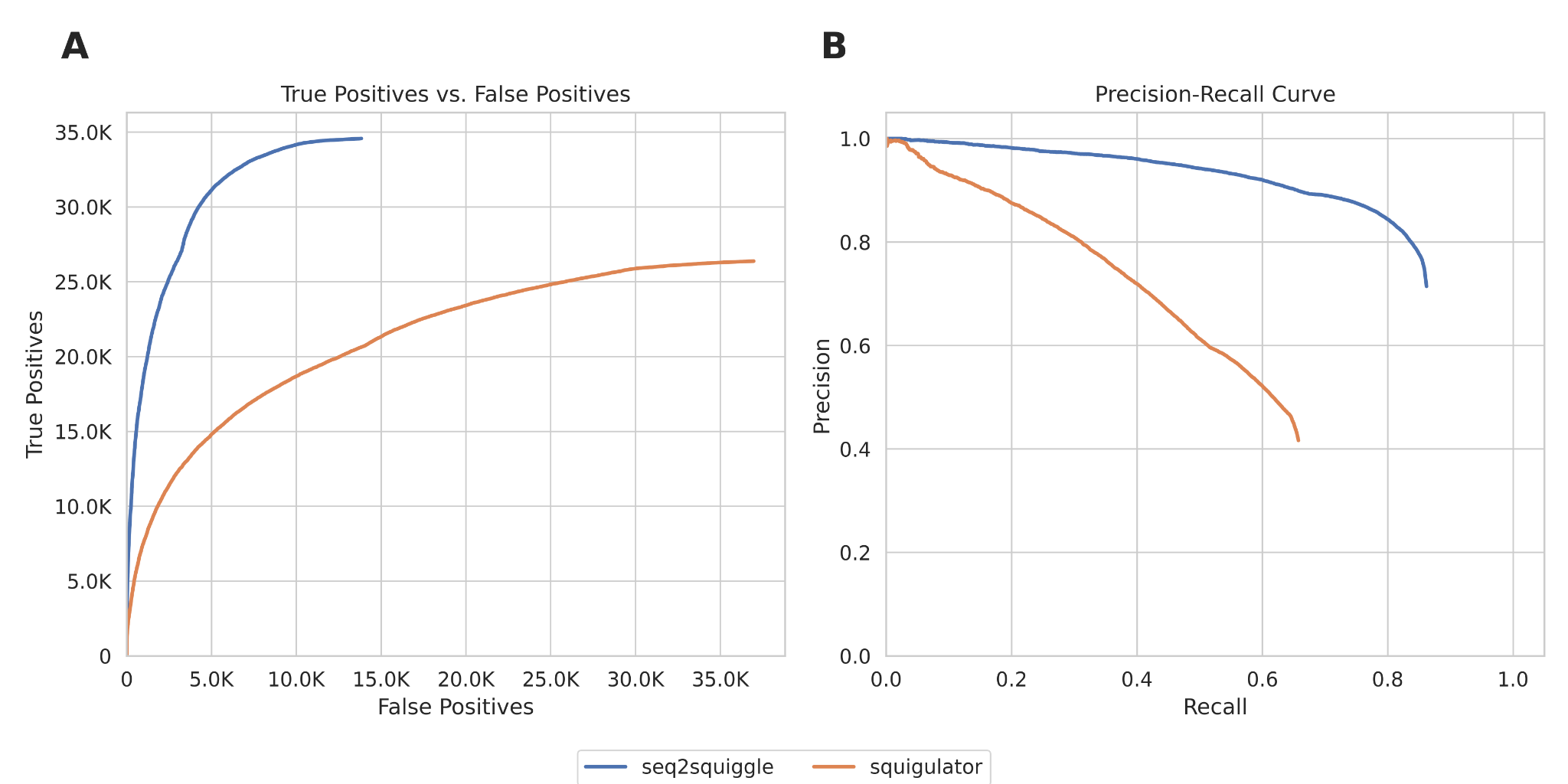


Supplementary Figure 6. Accuracy evaluation of SNP and Indel detection by Clair3 comparing squigulator and seq2squiggle on R10.4.1 data. (A) Receiver Operating Characteristic (ROC) curve illustrating the relationship between the total number of false positives and true positives. (B) Precision-Recall curve showing the precision and recall performance.


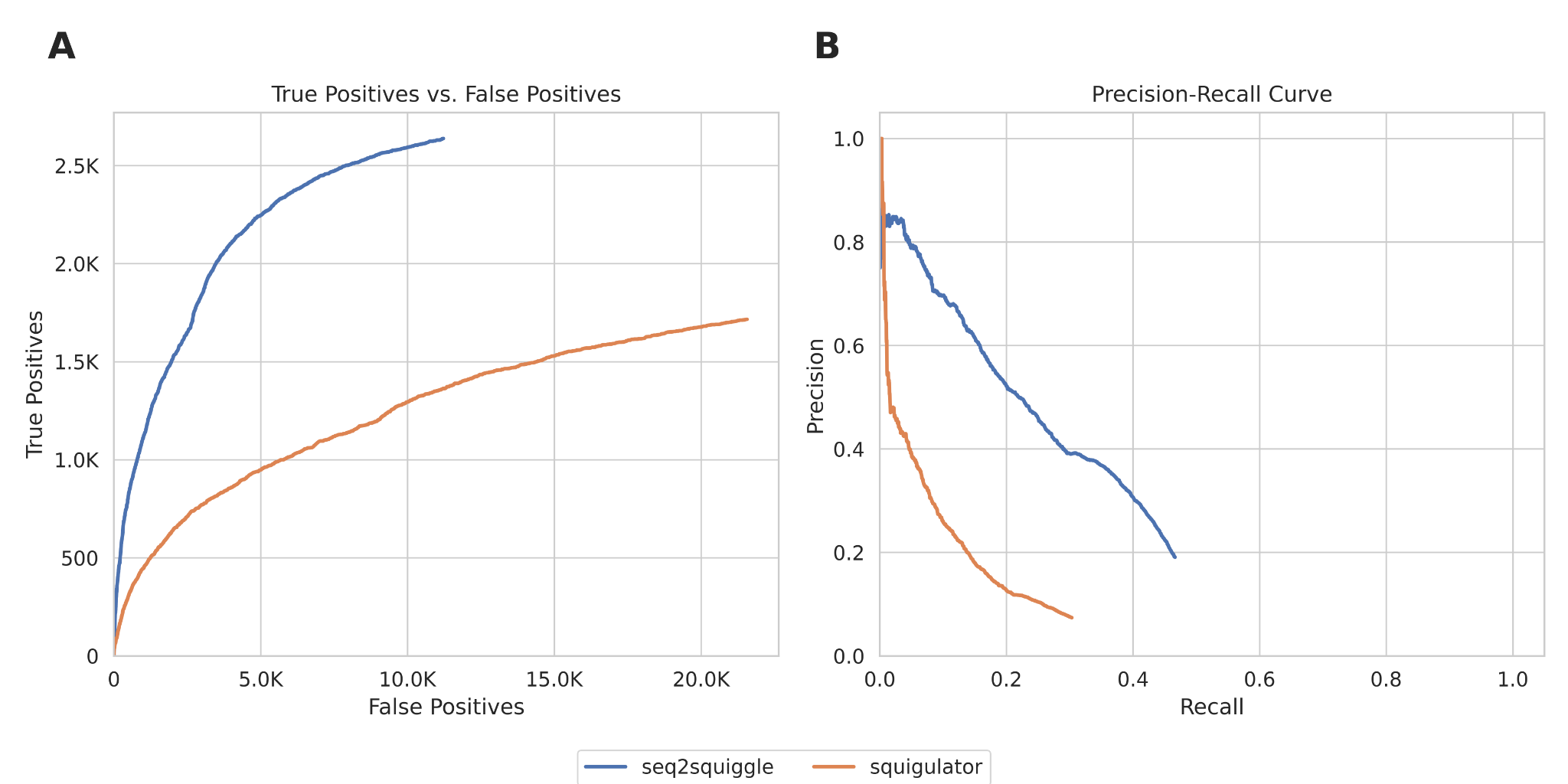


Supplementary Figure 7. Accuracy evaluation of Indel detection by Clair3 comparing squigulator and seq2squiggle on R10.4.1 data. (A) Receiver Operating Characteristic (ROC) curve illustrating the relationship between the total number of false positives and true positives. (B) Precision-Recall curve showing the precision and recall performance.


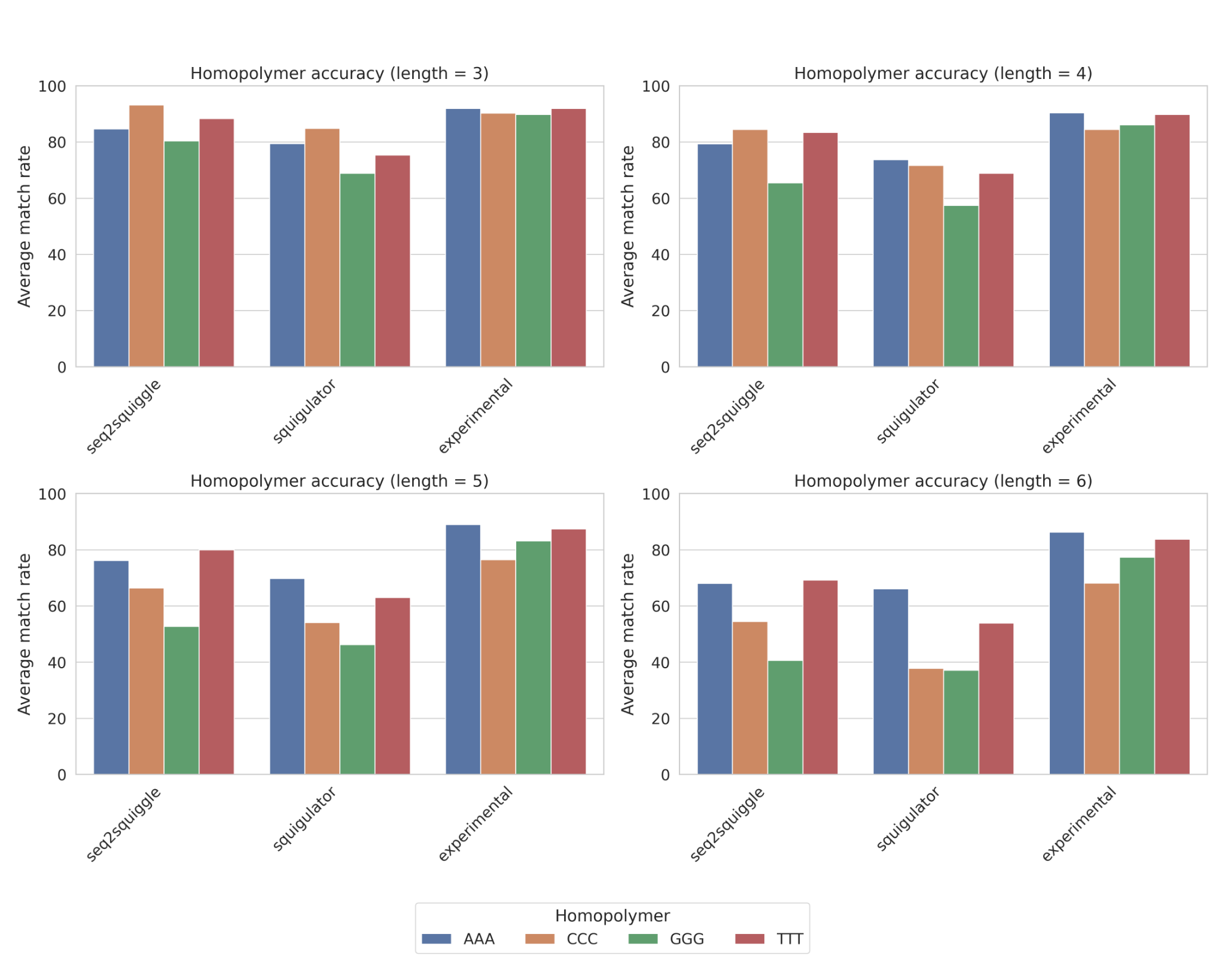


Supplementary Figure 8. Average match rate for different homopolymer lengths on the R.10.4.1 human dataset. Squigulator and seq2squiggle were executed in genome mode.


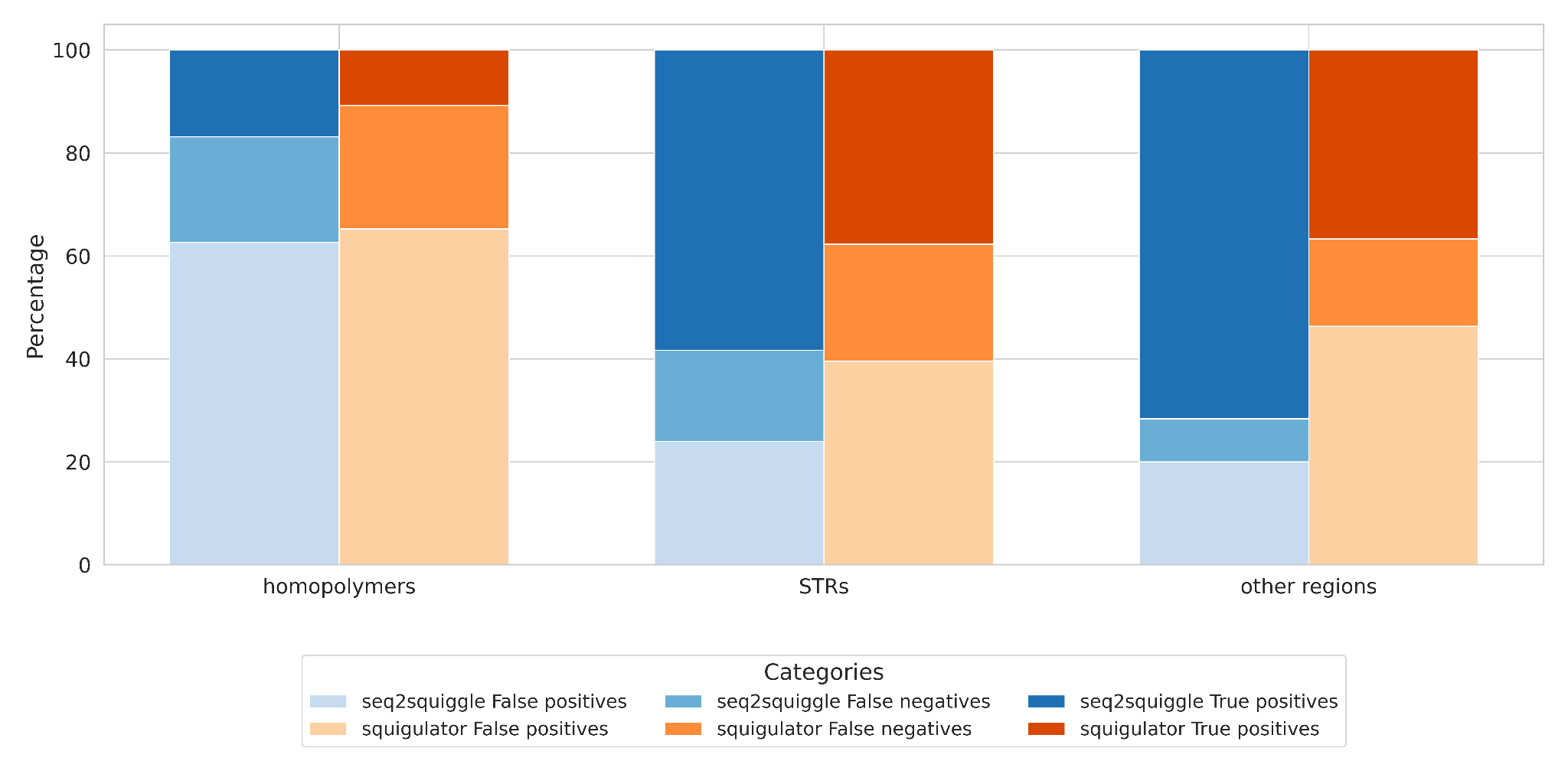


Supplementary Figure 9. Distribution of false positives, false negatives, and true positives across various genomic regions on the R10.4.1 human dataset. The relative proportions of these metrics are presented for squigulator (orange) and seq2squiggle (blue) in homopolymer regions, short tandem repeat regions, and other genomic areas. Homopolymer regions are defined as sequences with at least five consecutive identical bases, while short tandem repeat regions are identified using Tandem Repeats Finder.


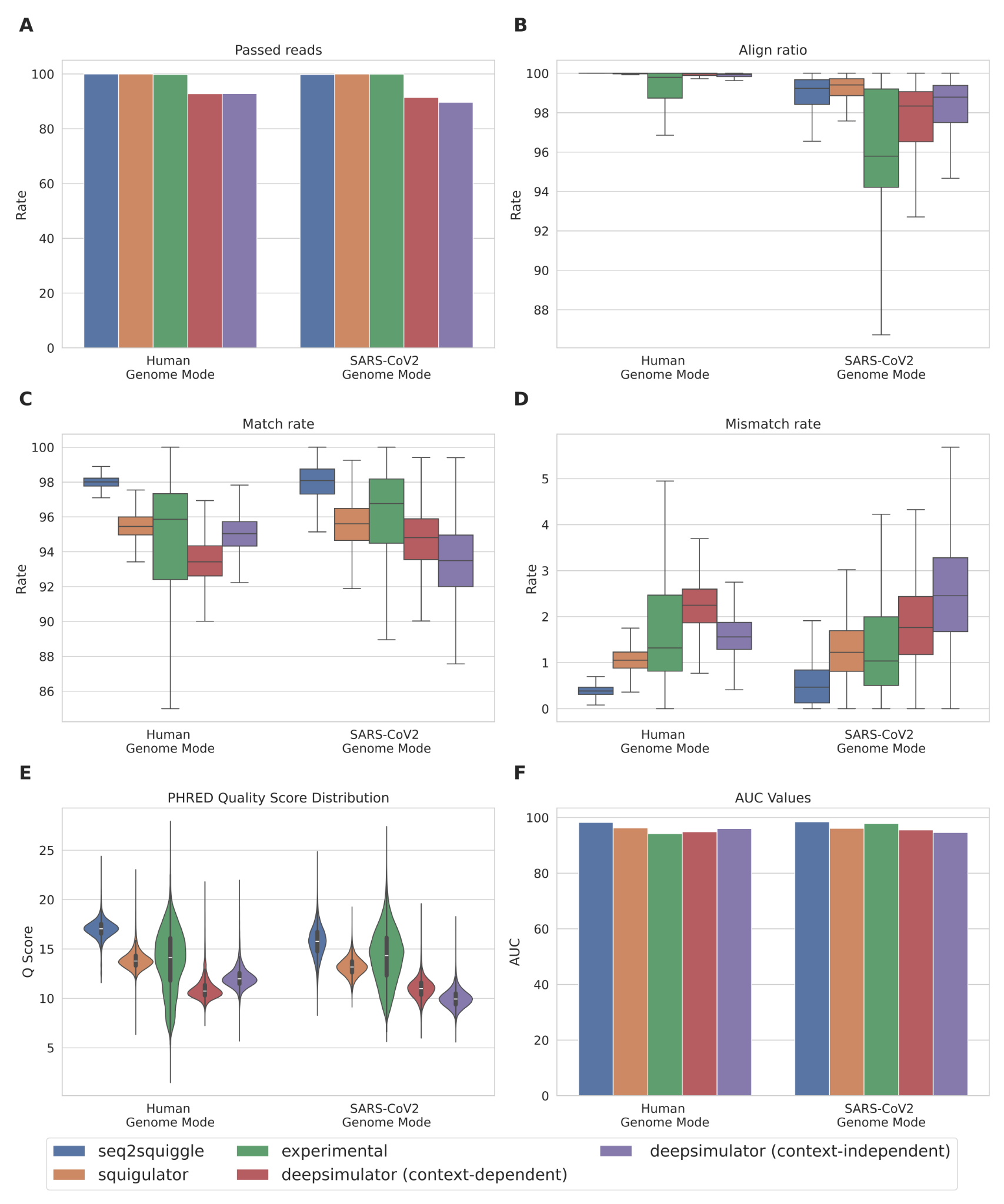


Supplementary Figure 10. Performance comparison of seq2squiggle (blue), squigulator (orange), experimental data (green), DeepSimulator in context-dependent mode (red), and DeepSimulator in context-independent mode (violet) across multiple R.9.4.1 datasets and several performance metrics. (A) Proportion of successfully aligned reads to the reference genome. (B) Distribution of aligned bases to the reference genome. (C) Distribution of match rates. (D) Distribution of mismatch rates. (E) Distribution of PHRED quality scores (F) Area under the curve (AUC) values for match rates sorted by average PHRED scores.


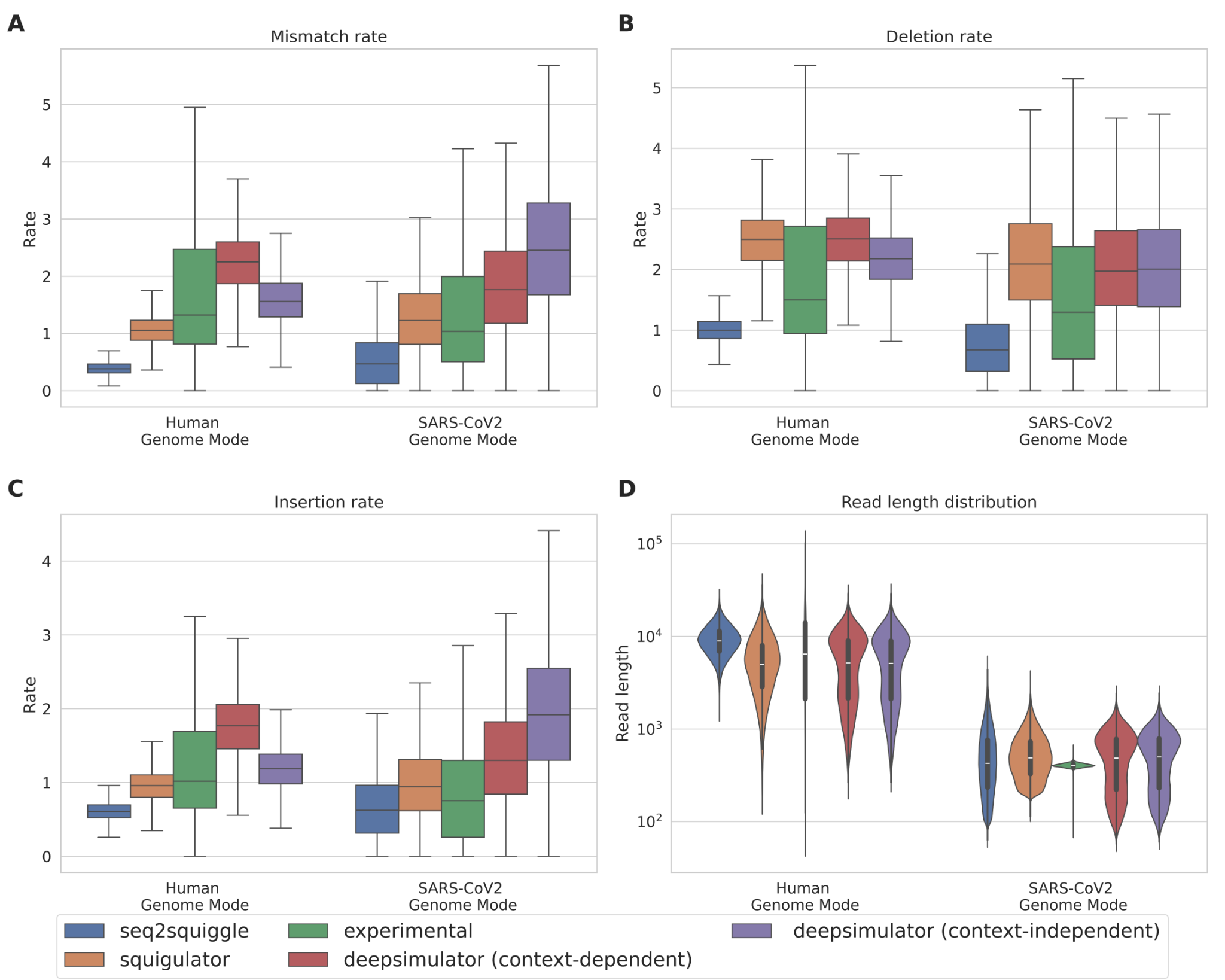


Supplementary Figure 11. Performance comparison of seq2squiggle (blue), squigulator (orange), experimental data (green), DeepSimulator in context-dependent mode (red), and DeepSimulator in context-independent mode (violet) across multiple R.9.4.1 datasets and several performance metrics. (A) Distribution of mismatch rates. (B) Distribution of deletion rates. (C) Distribution of insertion rates. (D) Distribution of read lengths.


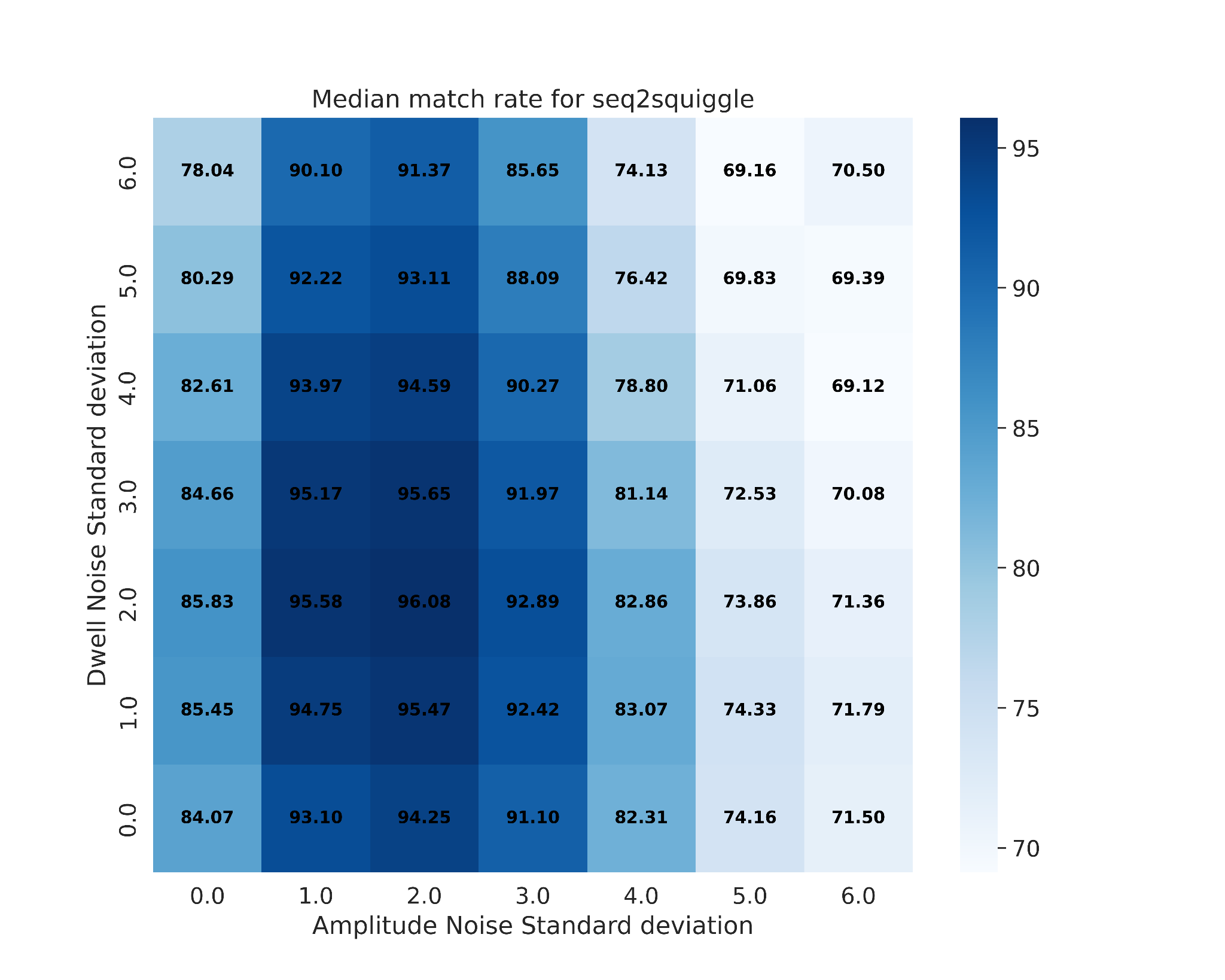


Supplementary Figure 12. Heatmap showing the median match rate of *seq2squiggle* across varying levels of amplitude noise (x-axis) and event length noise (y-axis) on the R10.4.1 D.melanogaster dataset. *Seq2squiggle* simulations were performed without the noise sampler and duration sampler, applying a normal distribution for both event length and amplitude noise. Values were excluded if fewer than 100 reads were successfully aligned.


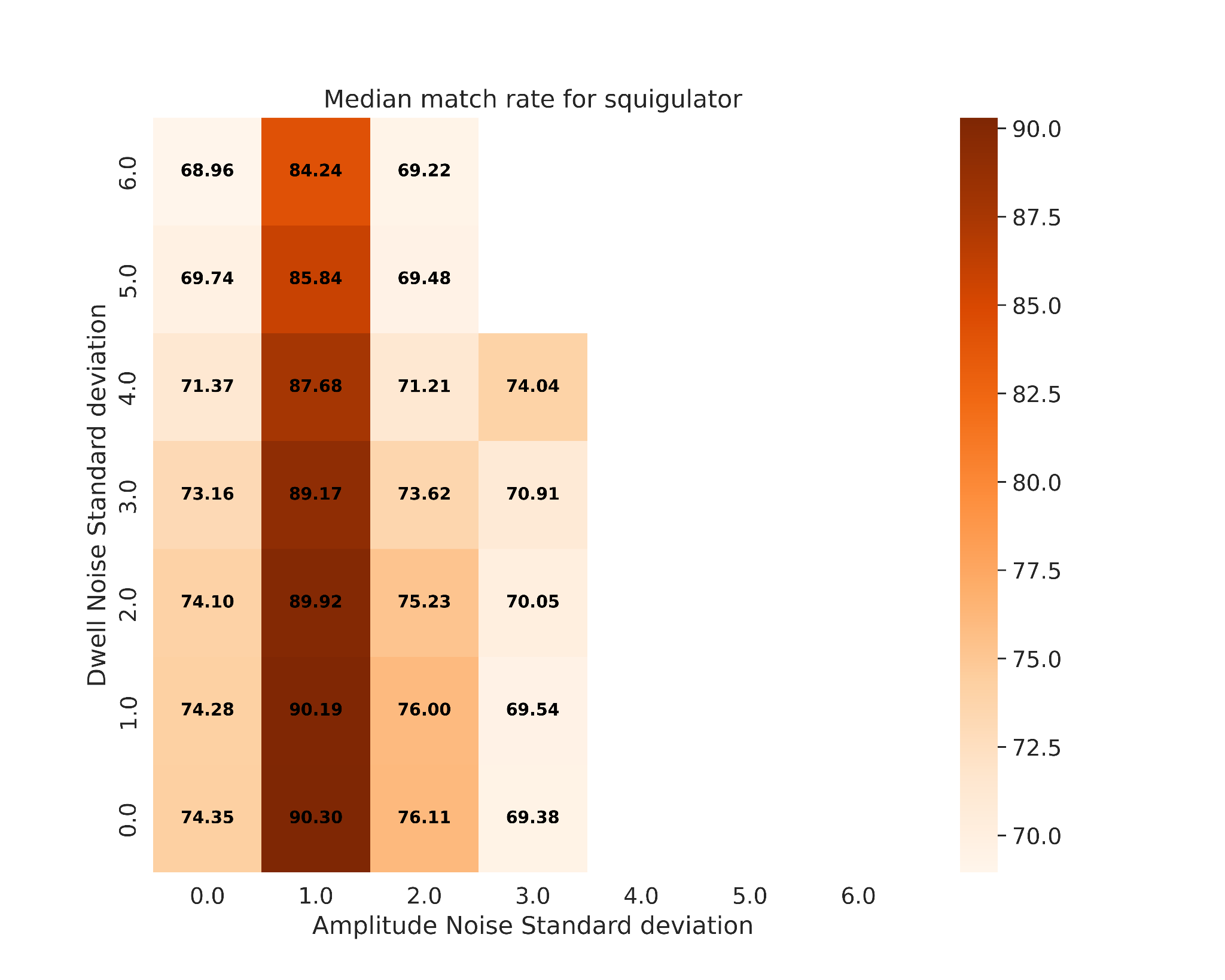


Supplementary Figure 13. Heatmap showing the median match rate of squigulator across varying levels of amplitude noise (x-axis) and event length noise (y-axis) on the R10.4.1 D.melanogaster dataset. Values were excluded if fewer than 100 reads were successfully aligned.

Supplementary Table 1 - Overview of datasets used for training and evaluation, including dataset name, number of reads, flow cell chemistry, corresponding website, dorado model used for basecalling, and reference sequence used for alignment with minimap2.

| Dataset name | # of total reads | Chemistry | Website | Dorado Basecalling model | Reference sequence |
| --- | --- | --- | --- | --- | --- |
| Drosophila melanogaster | 229,767 | R10.4.1 Kit 14 (4kHz) | <https://labs.epi2me.io/open-data-drosophila/> | dna_r10.4.1_e8.2_400bps_sup@v4.1.0.cfg | Genome Assembly Release 6 plus ISO1 MT |
| Human HCT116 (non-methylated) | 5,149,102 | R10.4.1 Kit 14 (4kHz) | <https://hasindu2008.github.io/f5c/docs/r10train> | na_r10.4.1_e8.2_400bps_sup@v4.1.0.cfg | hg38 (no alt contigs) |
| E. coli | 81,037 | R10.4.1 Kit 14 (5kHz) | <http://ftp.sra.ebi.ac.uk/vol1/run/ERR138/ERR13848445/> | dna_r10.4.1_e8.2_400bps_sup@v5.0.0 | NC_004431.1 |
| Human NA12878 | 500,000 | R9.4.1 Kit 10 (4kHz) | <https://slow5.bioinf.science/na12878_prom_sub_slow5> | dna_r9.4.1_e8_sup@v3.3 | hg38 (no alt contigs) |
| SARS-CoV-2 SP1 | 1,382,016 | R9.4.1 Kit 10 (4kHz) | <https://slow5.bioinf.science/SP1-raw-mapped> | dna_r9.4.1_e8_sup@v3.3 | Wuhan-Hu-1 (MN908947.3) |

Supplementary Table 2 - Model hyperparameter for seq2squiggle

| Parameter | Value |
| --- | --- |
| DNA Embedding dimension | 64 |
| Pre-Net Layers | 1 |
| Pre-Net Hidden | 64 |
| Encoder FFT Layers | 2 |
| Encoder FFT Hidden | 64 |
| Encoder Attention Heads | 8 |
| Encoder feed forward upwards projection size | 256 |
| Decoder FFT Layer | 2 |
| Decoder FFT Hidden | 64 |
| Decoder Attention Heads | 8 |
| Decoder feed forward upwards projection size | 256 |
| Duration Sampler Hidden | 64 |
| Noise Sampler Hidden | 64 |
| Dropout | 0.1 |
| Batchsize | 512 |
| Maximum Learning Rate | 0.0005 |
| Warmup Ratio | 0.01 |
| Learning Rate schedule | Linear warmup followed by cosine decay |
| Gradient clipping value | 1.0 |
| Optimizer | Adam |
| Total number of parameters | 219,780 |

Supplementary Table 3 - Average runtime and memory usage for generating 50,000 human R9.4.1 reads in Genome mode using *seq2squiggle*, squigulator, DeepSimulator in context-dependent (CD) mode, and DeepSimulator in context-independent (CI) mode. All tools were run with 64 threads, with seq2squiggle using a single A100 GPU.

| Tool | seq2squiggle | squigulator | DeepSimulator (CD) | DeepSimulator (CI) |
| --- | --- | --- | --- | --- |
| Runtime (in hour, minutes, seconds format) | 0:18:16 | 0:00:40 | 0:44:46 | 0:26:30 |
| max_rss (in MegaByte) | 131906.89 | 484.49 | 782934.09 | 79633.39 |
| max_vms (in MegaByte) | 3543044.52 | 1001.27 | 1010345.90 | 230337.08 |
| max_uss (in MegaByte) | 17981.64 | 481.41 | 64351.77 | 3944.42 |
| max_pss (in MegaByte) | 18610.69 | 481.91 | 75429.84 | 5118.72 |
| io_in (in MegaByte) | 32.19 | 240.82 | 1114.23 | 1920.11 |
| in_out (in MegaByte) | 1683.42 | 4356.11 | 6412.68 | 20789.20 |
| mean_load | 175.83 | 266.41 | 4419.27 | 314.18 |
| cpu_time | 1937.90 | 109.34 | 119928.10 | 5023.33 |

Supplementary Table 4 - Area Under the Curve (AUC) of match rate sorted by PHRED score for the default implementation *seq2squiggle* using learned noise modules and *seq2squiggle* using static normal distribution for noise (mean=9.0, std=4.0) and event-length (mean=0.0, std=1.0) sampling. The comparison is made across four different noise modes.

| Noise in amplitude domain | Noise in event-length domain | learned noise modules | static noise modules |
| --- | --- | --- | --- |
| **✓** |  | 93.93 | 93.76 |
|  | **✓** | 83.71 | 84.05 |
| **✓** | **✓** | 93.24 | 94.61 |
